# Cancer Stem Cells in Tumor Microenvironment of Adenocarcinoma of the Stomach, Colon, and Rectum

**DOI:** 10.3390/cancers14163948

**Published:** 2022-08-16

**Authors:** Jose Francisco Islas, Adriana G. Quiroz-Reyes, Paulina Delgado-Gonzalez, Hector Franco-Villarreal, Juan Luis Delgado-Gallegos, Elsa N. Garza-Treviño, Carlos A. Gonzalez-Villarreal

**Affiliations:** 1 Biochemistry and Molecular Medicine Department, School of Medicine, Universidad Autonoma de Nuevo Leon, Monterrey 64460, Mexico; 2 Althian Clinical Research, Monterrey 64000, Mexico; 3 UDEM Vicerrectoria de Ciencias de la Salud, Universidad de Monterrey, San Pedro Garza García 66238, Mexico

**Keywords:** cancer stem cells, gastrointestinal adenocarcinomas, colon cancer and gastric cancer

## Abstract

**Simple Summary:**

Gastrointestinal cancers have a high mortality rate worldwide, and the progression of the disease is related to cancer stem cells. Until now, its relationship with the microenvironment has been poorly understood. We describe the molecules and different pathways activated during this interaction and the new targeting therapies for cancer cells and microenvironment modulation. This approach could impact the way gastrointestinal cancers are managed.

**Abstract:**

Gastrointestinal adenocarcinomas are one of the world’s deadliest cancers. Cancer stem cells and the tissue microenvironment are highly regulated by cell and molecular mechanisms. Cancer stem cells are essential for maintenance and progression and are associated with resistance to conventional treatments. This article reviews the current knowledge of the role of the microenvironment during the primary establishment of gastrointestinal adenocarcinomas in the stomach, colon, and rectum and its relationship with cancer stem cells. We also describe novel developments in cancer therapeutics, such as targeted therapy, and discuss the advantages and disadvantages of different treatments for improving gastrointestinal cancer prognosis.

## 1. Introduction

Cancer is a multi-step process during which cells acquire an untamed ability to grow, proliferate, and (most times) differentiate, ultimately leading to either improper organ growth or the establishment of inadequate cells in locations where they contribute negatively to the body homeostasis, causing (amongst other things) high levels of inflammation [1,2,3,4,5]. Colorectal and gastric cancers are the most prevalent cancer in the digestive track. They represent the third and fifth leading causes of cancer-related deaths. With over 500,000–990,000 new cases worldwide, they typically have a five-year survival rate (mainly due to late diagnosis) [6,7,8].

**Figure 1:**
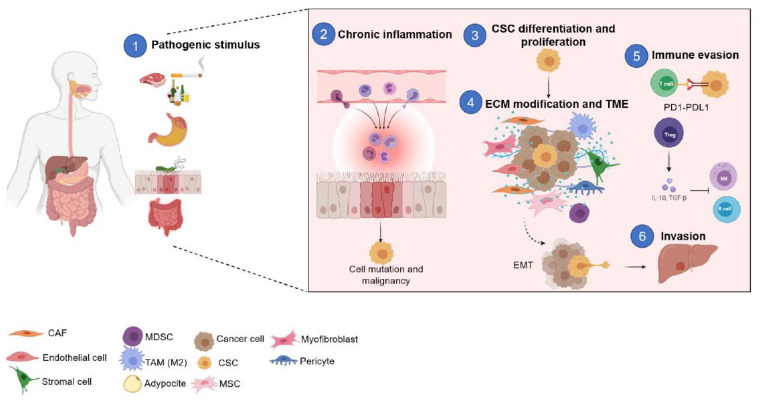
**Impact of microenvironment during the development of gastric and colon cancer.** Risk factors such as smoking, red meat, high alcohol intake, bacterial infections, and colitis could lead to chronic inflammation of the gastrointestinal tract, inducing cell malignization. All of these changes in the microenvironment induce cancer stem cell (CSC) differentiation and proliferation, with the contribution of cells that are attracted by inflammation and CSC. In addition, CSCs induce immune evasion and take advantage of immune regulatory mechanisms such as T regs, which allow epithelial-mesenchymal transition (EMT), invasion, and metastasis.

Smoking, alcohol consumption, and obesity are some of the most likely risk factors associated with this disease. Those factors can alter the tumor microenvironment modulating the most relevant tumor-promoting functions: inflammation, angiogenesis, metabolism, and epithelial-mesenchymal transition (EMT) [7,9,10]. Cells presented in the tumor microenvironment secrete different growth and differentiation factors, leading to an imbalance between cancer cells and cancer stem cell (CSC) self-renewal and differentiation. In particular, CSCs are a small subpopulation inside the tumor heterogeneity with clonal and tumor initiation ability and also have been related to chemoresistance [11,12]. Together, these components build a microenvironment that favors cancer cell proliferation and dissemination, providing them protection from apoptosis and host immune surveillance [13]. Thus, this study describes the interactions between CSCs and the tumor microenvironment (TME) which allow for cancer progression and cancer cell resistance. Gastric and colorectal cancers are related diseases that share risk factors, initial features, and pathophysiological mechanisms with multiple similarities that affect the TME and CSC (as shown in Figure 1).

## 2. Generalities and Risk Factors by Gastrointestinal Cancers

Most colorectal tumors develop slowly through a series of morphological, histological, genetic, and epigenetic changes. They are generally asymptomatic until they reach a considerable size, thus increasing the risk for adenomas to develop into colorectal cancer (CRC) [14,15]. Several factors increase the risk of developing this disease. Among the most studied were a red and/or processed meat diet, high alcohol intake, tobacco use, and sedentarism [16]. Other risk factors associated with the development of CRC are non-modifiable, such as a family history of colorectal polyps, CRC, lynch syndrome, inflammatory bowel disease, type 2 diabetes, and racial and ethnic backgrounds [17]. CRC typically develops from focal changes within benign, precancerous polyps. These polyps are localized growths or aggregations of abnormal cells within the intestinal mucosa that protrude into the intestinal lumen [17].

Whether sporadic or hereditary, gene mutations also increase the risk for CRC. Some mutations in DNA mismatch repair (MMR) genes, such as MLH1, MSH2, PMS2, and adenomatous polyposis coli gene (APC), are uncommon and have a low prevalence in hereditary cancer [18]. The chromosomal instability pathway is observed in 65–70% of sporadic cancers. The first mutations develop within the APC gene cell division and the next mutations develop in the KRAS oncogene, which affects cell differentiation, growth, motility, and survival. This causes a loss of function of the p53 gene, which acts as a transcription and apoptosis regulator that results in carcinogenesis [19]. Another epigenetic alteration seen in SSP-based CRC is aberrant gene promoter region hypermethylation, which inhibits gene transcription, regulating growth-promoting genes, bone morphogenic protein 3, and n-Myc downstream-regulated gene 4 (NDRG4) [17,19,20]. 

CRC can be classified among subtypes based on transcriptomic profiles. Moreover, every molecular subtype presents a different microenvironment [21,22]. These are summarized in Table 1.

Common risk factors for gastric cancer (GC) include older age, male sex, tobacco smoking, radiation, and family history. Other factors are obesity, helicobacter pylori infection, gastroesophageal reflux disease, and diet (such as a high intake of salty foods or low vitamin A and C intake), nitrosamines, chemicals, smoked food, and high alcohol consumption [7]. In particular, high salt intake and tobacco are associated with increased incidence and mortality of GC [26]. The carcinomas are a consequence of a multistep process starting from chronic inflammatory through a sequence of atrophic gastritis, metaplasia, low-/high-grade dysplasia, and finally cancer. Gastritis is caused by chronic inflammation and is commonly regarded as a point of no return for carcinogenesis [27]. This results from persistent infection, which may evolve chronic atrophy gastritis, and subsequent changes in the gastric mucosa. *Helicobacter pylori* is the most prevalent bacterial pathogen currently classified as a group one carcinogen by the World Health Organization (definite carcinogen) [28].

Environmental and genetic factors can lead to cancer initiation by mutations that improve stemness and invasion. CSC are tumor cells that have the main properties of self-renewal, clonal tumor initiation capacity, and clonal long-term repopulation potential associated with the initiation, progression, and therapy resistance of cancer [29]. CSCs interaction with the microenvironment preserves their phenotypic plasticity, protects them from immune system elimination, and facilitates the growth and expansion of cancer cells [30].

## 3. Cancer Stem Cells and Tumoral Microenvironment

CSC exhibit several remarkable abilities mentioned elsewhere. However, since they can derive from normal stem cells, they might also arise through dedifferentiation of mature somatic cells to reacquire stem cell characteristics. Studies have shown that CSCs are also responsible for therapy resistance by developing an increased expression of drug transporters, maintaining a slow dividing state, and efficient DNA damage repair mechanisms [31]. The identification of CSCs is based on markers found in normal stem cells, and these vary according to the type of cancer. CD44, CD24, and CD133 are usually common in gastrointestinal cancers. NANOG, OCT4, SOX2, and KLF4 transcription factors are considered critical of stem self-renewal and pluripotency regulators, mediating tumor proliferation and differentiation [27]. Also, overexpression of components from signaling pathways such as JAK/STAT, WNT, NOTCH, SHH, PI3K/phosphatase and Tensin homolog, and NF-κB signaling pathways are involved in renewal, differentiation, and uncontrolled proliferation of CSC [32]. 

Inside the tumor, the CSC niche can be a product of numerous factors specific to the TME and considered important factors extrinsically influencing tumor heterogeneity. These CSC can affect the tumor’s aggressiveness or invasion, modulating the normal growth and development of resident stem cells [30,33]. In turn, the accumulation of key mutations appears to begin specifically within the CSC compartment. Related EMT markers TGF-β1, TWIST, SNAIL, SLUG, vimentin, and CD44 were upregulated, and CDH1 mRNA levels were decreased in the gastric mucosa of patients with dysplasia or early GC in comparison with controls. A variant of CD44, a hyaluronic acid receptor (CD44v8-10), was identified as the predominant CD44 variant expressed in GC cells and contributes to tumor progression, possibly by enhancing oxidative stress defense [30]. 

Another proposed origin of CSCs is derived from the transformation of bone marrow-derived mesenchymal stem cells (BM-MSCs) in the GC, which served as primary cell components contributing to tumor progress, migration, and angiogenesis [34]. It has been observed that after infection and inflammatory stimulation of BM-MSCs, they migrate to the gastric epithelium and participate in tissue repair of the gastric mucosa [35]. 

A tumor’s diverse cell population tends to be erratic rather than regulated. However, CSC within the tumor is exposed to several biomolecules lead to an imbalance between CSC self-renewal and differentiation [35]. In CRC, MSC-derived exosomes are a double-edged sword in cancer development, metastasis, and invasion [36,37]. Tumor-associated stromal cells are key contributors to the tumor microenvironment, these arise from distinct sources, consisting of the basement membrane, non-malignant cells (fibroblasts, BM-MSC, adipocytes), immune cells (macrophages, lymphocytes), extracellular matrix, and vasculature (endothelial cells, pericytes) [38]. Thus, the CSC microenvironment produces factors that stimulate CSC self-renewal, induce angiogenesis, and recruit immune cells and other stromal cells that secrete additional factors to promote tumor cell invasion, as shown in Table 2. 

## 4. Crosstalk between Oncogenic Signaling and Metabolic Pathways of CSC: Role of Stroma-Derived Chemokines in the Local Invasion of Primary Tumor

Inflammation and tissue damage attract cells that cooperate with tumor TME as immune cells, stromal cells, adipocytes, and ECM components, whose main role is inhibiting apoptosis, protection of tumoral cells, promotion of proliferation, immune evasion, and invasion [10,47]. ECM is formed by a variety of substances, including collagen, elastin, laminin, fibronectin, and modulators such as metalloproteinase (MMP), which cleaves the ECM components and is crucial for tissue remodeling [48]. The persistent stimulus of inflammation in the tissue affects the homeostasis of cells, matrix, and cytokines. This finally leads to fibrosis and remodeling of ECM, which contributes to the establishment of tumors [49]. 

One example of this process is when the GC microenvironment becomes infiltrated by CAFs and TAMs, leading to excessive fibrosis [50]. The stimulus broadens the range of proinflammatory cytokines such as IL-6, TGF-β, FGF (fibroblast growth factor), TNF-α, and IL-1β that promote EMT [51]. Also, these cells induce fibrosis in ECM, which is associated with a worse prognosis in GC. In colitis-associated cancer, Liang et al. demonstrated that sphin-gosine-1-phosphate (S1P) induces an amplifying loop of SIPR1 and NF-kβ/IL-6/STAT3 [52]. Interleukins such as IL-6 and gp130-related are the main activators of the JAK2/STAT3 pathway in CSCs. In addition, IL-6 promotes the survival of pre-malignant intestinal epithelial cells, which then transforms into tumoral cells [53,54]. CAFs enhance GC cell migration and EMT through the secretion of IL-6 [50]. Once activated, the STAT-3 signaling pathway induces Tlr2 gene transcription in the gastric epithelium, which after overexpression, promotes proliferation and inhibits gastric epithelial cell apoptosis [55]. Tumor cells take advantage of STAT-3 by increasing immune evasion by inhibiting the maturation of dendritic cells (DC), which activate the adaptive immune response [56]. In recent years, WNT5a signaling in CAF has been implicated in tumor progression. In turn, overexpression of β-catenin and WNT5a indicates a poor prognosis since they promote cell growth, migration, invasion, and EMT of digestive tract tumors [39]. In addition, CSCs promote tumor infiltration of TAM through the CD44 receptor, which is upregulated by miR-328 suppression. Moreover, MSCs present an immunomodulatory role on lymphocytes B, T, dendritic cells, macrophages, and MDSCs. Therefore, MSC could affect CD4+ T cell migration and differentiation, T helper 17 homeostasis modulation, and response [51]. In addition, STAT3 mediated TWIST expression and EMT can be activated by EGF/EGFR [52]. 

Chemokines act by interacting with specific G protein-coupled receptors, and chemokines from TME can facilitate tumor progression or remodeling of the tumor niche by signal transduction [57]. Thus, these proteins may play a crucial role in the pathogenesis of CRC, and GC, as shown in Table 3. However, the high heterogeneity of the cell context limits transcription factors potential to induce gene expression on CSCs and their changes to improve tumor progression [58]. It is being proposed that transcription factors such as the Yes-Associated Protein (YAP)/Transcriptional Co-activator with PDZ-binding Motif (TAZ) pathway lead to epithelial phenotype repression and participate together with factors of the SNAIL family, such as TWIST and Zeb, which are the main regulators of EMT [59]. YAP overexpression has been reported in GC, and CRC; protein levels are associated with poor prognosis, tumor stage, and metastasis [60,61,62].

Inflammation and tissue damage are a potent chemokine source; both processes recruit cells that cooperate with tumor TME as immune cells, stromal cells, adipocytes, and ECM components. The main role of these cells is to inhibit apoptosis, protect tumoral cells, promote proliferation, and immune evasion and invasion [10,47]. 

Gastrointestinal epithelial cell infection by *H. pylori* and the expression of CXCL1 gastric cancer cells is necessary to stimulate the migration of bone marrow-derived stromal cells (BMDC) by CXCR2 signaling; moreover, the expression of CD271 by BMDC is related with invasion and worse prognosis [63]. Tumor-associated macrophages (TAM) are associated with tumor stage and metastasis. There are two types of macrophage differentiation: M1 macrophages that produce type I proinflammatory cytokines such as IL-1β, IL-1α, IL-12, TNF-α, and GFAP, and M2 macrophages that produce type II anti-inflammatory cytokines such as IL-4, IL-6, and IL-10, related to the pro-tumorigenic activity. The change from one phenotype to another depends on the TME, and the high infiltration of M2 is related to a worse prognosis [64]. Chronic gastritis induced by *H. pylori* infection is associated with 90% of gastric cancer cases. It has been reported that this infection causes atrophy of acid-secreting parietal cells (PC), which increases CD44+ stem cell proliferation in the gastric isthmus by pERK [26,65]. Some gastrointestinal hormones, such as gastrin or neurotransmitters and acetylcholine (Ach), may also play unique roles in the antral stem cell niche. Gastrin is secreted from G cells and is responsible for HCl secretion in parietal cells (PCs). These cells reside near the antral isthmus region [66]. Gastrin also induces the expression of EGFR ligands as heparin-binding EGF and trefoil family factor 2, which activate the cholecystokinin receptor (CCK-BR), and thus, motility, secretion, migration, and proliferation of gastric cells [67]. Moreover, MMPs and tissue inhibitors of metalloproteinases (TIMPs) decrease E-Cadherin and ECM degradation cell change interactions and paracrine signals, helping malignant transformation [68]. For example, *H. pylori* increase IL-21 expression in infected gastric mucosa and promote gelatinases, MMP-2, MMP-7, and MMP-9 synthesis through NF-kβ in activated B cells [68]. Some other proteins increase their expression during GC, such as phosphoglycerate kinase 1 (PGK1), CXCR4, CXCL12, and β-catenin [46], which promote EMT. In addition, CAFs secrete multiple proinflammatory cytokines such as TGF-β1, IL-β1, IL-6, IL-33, ROS, C-X-C chemokine receptor (CXC), MMPs, lysyl oxidase, miR-21, TNF-α, and αSMA [46]. All of these factors contribute to tissue fibrosis, and later fibrogenesis activates ECM remodeling. In addition, fibrogenesis activates the chemoresistance-inducing factor SNAIL in epithelial cells, promoting proliferation and inducing drug resistance [69]. Table 4 includes the MMPs present in GC and CRC. 

**Table 3:** Chemokines in the tumor microenvironment.

Chemokine	Receptor/Pathway	Gastrointestinal Cancer Improvement	Reference
IL-6	IL-6R, activation of JAK2-STAT3	Promotes proliferation and EMT	[53]
WNT5a	Frizzled receptor, activation of WNT/β-catenin pathway	Promote cell growth, migration, invasion and EMT	[70]
PGK1	Upregulates CXCR4 and β-catenin	Promotes EMT and metastasis	[46,71]
IL-21	IL-21R, increase NF-kβ in activated B cells	promotes gelatinases, MMP-2, MMP-7, and MMP-9, and EMT	[68]
Gal-1	Prch, activation of Hedgehog signaling	Promote tumor invasion and EMT	[72]
POSTN	ERK and p38 pathways	Proliferation, invasion, and migration of cancer cells	[73]
CXCL12	CXCR4	Improves TME and angiogenesis, lamellipodia and filopodia, cell adhesion to ECM	[74]
CXCL8	CXCR1/CXCR2 regulates the expression of MMP-9, intracellular adhesion molecule (ICAM)-1, and E-cadherin.	Increased invasion, migration, and adhesion of cancer cells	[74]
CXCL1	CXCR2, higher levels of MMP-2 and MMP-9 and upregulation of Ras and STAT3	Tumor progression, increased migration, and invasive potential	[74]
CXCL5	ERK/SNAIL pathway	Progression and metastasis of GC and activation of pro-tumor neutrophils	[75]
CXCL7	CXCR2	Promote tumor growth and activation of pro-tumor neutrophils	[76]
CXCL9, CXCL10	CXCR3	Promotes metastasis to lymph nodes	[77]
CXCL8	CXCR2	Increase proliferation and invasive capacity	[76]
CXCL11	CXCR3 and CXCR7	Promotes cell growth and EMT	[78]
CXCL16	CXCR6	Enhanced the recruitment of tumor-infiltrating lymphocytes	[74]

## 5. Participation of Extracellular Matrix Components in Cancer Progression

The role of the ECM has been demonstrated in all stages of GC and CRC, from onset to metastasis [82,83]. However, it has been shown that some components of the protein family are shared by gastrointestinal tumors and regulate a key aspect in the early stages of tumor biology, participating in the regulation of CSC (which can be favored under the stimulation of some signs or changes in the microenvironment by ECM proteins) [84]. Intestinal epithelial cells express integrins in healthy and pathological circumstances. Integrins are categorized based on the preferred ligands; they bind to collagens for α1/α2 coupled to β1, laminins for α3/α6/α7 coupled to β1 or β4, tenascin for α9β1, and RGD-containing ligands (fibronectin, osteopontin, and vitronectin) for α5/α8 coupled to β1 and αV coupled to β1/β3/β5/β6/β8. Recent works address the altered expression of certain integrins and their involvement in GC and CRC progression [85]. For example, integrin V6 can be expressed by CRC cells, which can then inactivate TGFβ by integrin αvβ6 subsequently activating fibroblasts that promote tumor invasion [86]. CRC integrin αvβ8 expressed on tumor cells is reported as a crucial regulatory function during cell adhesion in the tumor microenvironment and supports the activation of TGF-β1 [87]. Several of these integrins, including integrin subunits β1, α6, β3, and β4, concentrated in healthy adult stem and progenitor cells, are also signs of CSCs. Another characteristic of CRC is mainly overexpression of collagen types I, VI, VII, VIII, X, XI, and XVIII. For example, increased expression of type 1 collagen promotes EMT and stem cell marker expression by activation of PI3K/AKT/Snail signaling pathway conducted by integrin α2β1 [88]. In addition, recent studies reported a higher expression of collagen XVII, which was significantly associated with the progression of cancer and by interaction with laminin-5 (Laminin-332), which is essential for epithelial cell migration and basement membrane attachment and, according to some studies, is a determinant for CCR initiation [89]. Figueiredo et al. reported that mutant E-cadherin conduces to an increase in β1 integrin expression associated with higher grade tumors and reduced overall survival of the GC patient [90]. Some of these proteins promote key CSC functions such as: (1) EMT; (2) immune surveillance modulation; (3) self-renewal/maintenance; and (4) metabolic reprogramming and matricellular proteins. These functions are also involved in the cellular mechanical response, such as mechano-sensor integrins, which are receptors for many proteins [91]. Table 5 summarizes the most important proteins associated with CSC and early stages and progression of colorectal and gastric cancer. In addition, these ECM proteins have been recently proposed as markers of early diagnosis and poor prognosis of gastrointestinal neoplasms associated with CSC signaling pathways. For example, Galectin-1 (Gal-1) is associated with *H. pylori* infection in GC, the increase in the expression of β-catenin, vimentin, and Hedgehog, and the decrease in E-cadherin expression [92,93]. In CRC, a higher expression in endothelial and tumor cells has been found in the stroma that promotes tumor invasion and progression. Although some reports have not found a coincidence with the tumor stage and progression in Gal-1 expression, more reports show that its expression is significant in the early and late stages of the disease. Therefore, it is an interesting target for gastrointestinal neoplasias [94]. The extracellular carbohydrate structure of Gal-1 produced by CAFs may interact with Integrin 1 to promote Gli1 expression, which may ultimately activate Wnt/-catenin signaling and result in the EMT process in GC cells [95,96]. To provide another example, a rigid, collagen-rich, or POSTN-rich ECM allows macrophage polarization to a pro-tumorigenic M2 phenotype. After recruitment, M2 macrophages activate several CSC survivals signaling pathways, including Src, NF-κB, STAT3/SOX2, and SHH. ECM can also affect the proliferation and activation of T cells, which are necessary to capture and kill CSCs. POSTN is overexpressed by CAFs and constitutes the primary tumor niche by supporting the proliferation of cancer cells through the ERK signaling pathway in GC [84]. 

**Figure 2:**
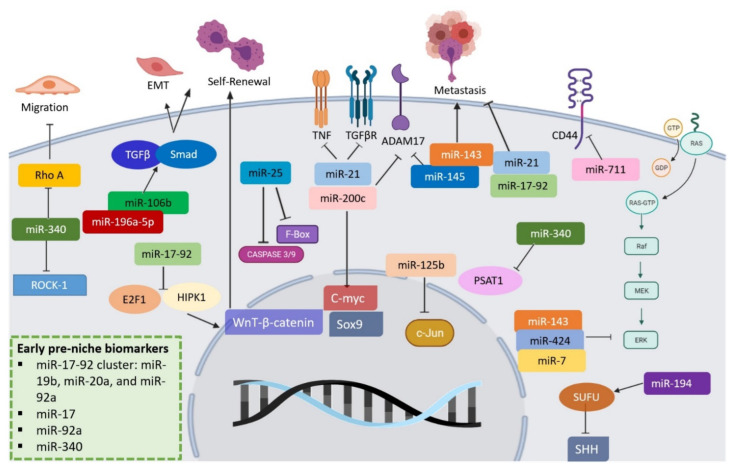
**Regulatory roles of miRNAs in gastrointestinal cancers.** miRNAs participate in the modification of several pathways that lead to or inhibit gastric and colon cancers. Some are present since cancer pre-niche and are related to EMT, migration, proliferation, invasion, and metastasis development.

Another important component in ECM are miRNAs. miR-206 downregulation in GC enhances GC stem cells (GCSCs) and carcinogenesis. At the same time, its overexpression suppresses GCSCs formation and associated tumorigenesis through ETS homologous factor (EHF) downregulation [112]. In GC, miR-17-92 members can help show metaplasia, but more importantly, they can be found at the early pre-niche stage serving a significant role as biomarkers [113]. In CRC, one of the most studied pathologies, circulating levels of miR-17 and miR-92a have been associated with early stages (primary tumor establishment) formation [3,114]. Having members of this cluster circulating (if further analyzed and quantified) can give us an idea of cancer staging in the gastrointestinal tract. Moreover, miR-340 is a well-studied miRNA known for its role in the early establishment of CRC [115,116]. The primary miRNAs that participate in several pathways and lead to or inhibit gastric and colon cancers are shown in Figure 2. 

In a bit more detail, in CD44 (+) cells, miR-106b enhances gastric stem cell (GCSC) traits such as EMT, self-renewal, and invasion through the modulation of the TGF-β/Smad signaling pathway [112]. miR-145 can further affect the stemness property of CD44 (+) cells, reducing levels of ADAM17 and SOX9, which regulate invasiveness. Loss of this regulation leads to tumor initiation via IL-6 inflammatory processes [117]. In addition, miR-196a-5p has been upregulated in CD44 + cells, and suppression led to less colony formation and invasion of GC stem cells, suggesting a significant role in EMT and invasion of this cell population [83]. miR-143, miR-145, miR-21, and miR-194 significantly distorted the epithelium of several cancers; these miRNAs relate to the target cell cycle (cdc25), PTEN signaling, FSCN1, ZEB2, and MDM2 [118,119,120,121,122]. 

Over 10 years, cluster miR-143 and miR-145 were shown to coregulate FSCN1 (early marker), taking part in metastasis [123,124]. Over time, this has led to identifying novel lymphatic directed biomarkers such as miR-135b-5p, miR-15b-5p, and miR-195-5p, correlated to pathological classifications. miR-145 seems to target c-Myc, inducing p53. Another interesting miRNA is miR-25, as its effects in GCs include viability, cell migration, and growth through an increase in cyclin D1. In colon cancer, miR-25 further decreases apoptosis by inhibiting caspases 3/9 [125]. In GC, miR-25 directly correlates with invasion by repression of the F-box and WD-40 domain protein 7 [126]. Tumor suppressor FBXW7 regulates degradation of the growth proteins, cyclin E, c-Myc, Mcl-1, mTOR, Jun, and Notch [126]. 

## 6. Treatment against Gastrointestinal Cancers

In usually incurable advanced tumors with metastases, patients get conventional therapy options such as surgery, radiotherapy, and chemotherapy [127]. However, chemoresistance has been reported as one of the main causes of treatment ineffectiveness, where CSCs overexpress P-gp, ABCG2, Bcl-2, and surviving overexpression [128]. Studies have reported that cell resistant to cisplatin, 5-FU or docetaxel showed hyperexpression of the stem cell marker proteins, CD44, CD133, ALDH1A1, NOTCH1, Oct4, and SOX2 showing increased migration and invasion, formation of spheroids, colonies, and tumorigenic [129]. This information, coupled with an increase in morbidity and mortality in gastrointestinal system cancers, forces us to continue researching new strategies to provide effective treatment that avoids cancer cell resistance.

New cancer therapies are directed at stimulating the immune system to eliminate CSC and revert immunotolerance. These immunotherapies have been studied in CRC, particularly in MSI/MMR status. Some immune components in the TME regulate tumor development. Thus, immune cells are a promising target for cancer immunotherapy [45]. Checkpoint inhibitors, such as the CTLA-4 and PD-1 inhibitors tremelimumab, and nivolumab, have presented limited activity. In addition, the Check-Mate-142 trial (Phase II) evaluated nivolumab and nivolumab plus ipimumab in patients with metastatic CRC with MSI-H/dMMR or MSS/pMMR status, showing improvement in patient survival [130]. Moreover, the FDA-approved nivolumab (Opdivo) combined with chemotherapy for initial treatment of patients with advanced or metastatic GC due to improvement in survival compared with placebo [131].

In the KEYNOTE-164 phase II trial, pembrolizumab monotherapy reached a one-year overall survival of 72% in patients pretreated with MSI-H mCRC [132]. In addition, the combination of pembrolizumab with cisplatin and 5-FU in a phase II clinical trial in subjects with recurrent metastatic GC (KEYNOTE-059/NCT02335411) increased survival, encouraging FDA approval of pembrolizumab in September 2017 as third-line therapy for recurrent or metastatic cancer [131]. Coadjuvant therapy with FOLFOX and PD-1/PD-L1 inhibitors achieved an objective response rate (ORR) of up to 50% and increased survival. In MSI-H/dMMR CRC, checkpoint inhibitors should be used after therapy with first-line drugs such as fluoropyrimidine, oxaliplatin, and irinotecan [130].

CAR-T cell therapies present efficacy against hematological malignancies and solid tumors, and recently CAR-T cells have been applied in gastrointestinal tumors against antigens such as epidermal growth factor receptor 2 (HER2), carcinoembryonic antigen (CEA), mucin 1 (MUC1) and epithelial cell adhesion molecule (EpCAM) to delay tumor growth in murine models [133]. MUC1-specific CAR-T cells effectively target MUC1-positive tumor cells. EpCAM-targeted CAR-T cells are being evaluated in clinical trials (phase I/II trials NCT02617134 and NCT02725125) to assess their safety and efficacy [131]. G17DT is a vaccine that neutralizes gastrin-17, a hormone required for the growth of GC cells. The phase II/III clinical trial (NCT00042510) showed that G17DT induced specific affinity against gastrin antibody, which inhibits tumor proliferation and metastasis [134,135].

Table 6 includes the new target molecules and pathways against CSC in gastrointestinal cancer.

## 7. Conclusions

Cancer is the final step from several cell changes due to genetic mutations and environmental epigenetic changes. As has been observed, gastrointestinal cancers share important characteristics from their beginning, mainly due to the development of a pre-niche that allows CSCs proliferation and establishment in tissue. Additionally, inflammation seems to be a central pillar in gastrointestinal cancer development, as cancer risk factors induce a state of chronic cytokine activation. Moreover, cancer cells use similar signaling pathways to survive. Signals from the pre-niche, such as ECM proteins, chemokines, and miRNAs derived from recruited cells, also enrich the tumor microenvironment and modulate the CSC response. Thus, the cooperation of all of these factors to develop tumorigenesis is necessary. It is not surprising that immunotherapy has been proposed as a strategy for managing gastrointestinal cancers, wherein a change in TME signals is introduced to eliminate CSC.

## Figures and Tables

**Table 1:** CRC subtypes.

Subtypes	Frequency	Characteristics	Mutations	TME Associated	Clinical Relevance	Reference
CMS*1 **Microsatellite instability immune**	14%	Diffuse immune infiltration ↓TGFβ inside TME	BRAF MSH6, RNF43, ATM, TGFBr2, PTEN	↓CAF ↑active adoptive immune response	Worse survival after relapse	[23,24,25]
**CMS2** **Canonical**	37%	Epithelial features, CIN, activated WNT and MYC signaling pathways	APC, KRAS, TP53, PIK3CA	↓CAF ↓immunogenic response		
**CMS3** **Metabolic**	13%	Epithelial and disregulation metabolic	APC, KRAS, TP53, PIK3CA	↓CAF ↑active adoptive immune response, affected glutaminolysis, lipidogenesis, damage of mechanisms DNA repair		
**CMS4** **Mesenchymal**	23%	upregulation of EMT, TGF-β activation, angiogenesis, stromal infiltration activation mesenchymals and complement	APC, KRAS, TP53, PIK3CA	Many CAF, inflammation promote EMT	Worse relapse-free and overall survival	
**Un-classified**	13%	Mixed phenotype of multiple CMSs or intratumoral heterogeneity				

* CMS, consensus molecular subtypes.

**Table 2:** Tumor microenvironment factors that improve CSC activity.

Cancer	Cell	Factor	Activity in CSC	Reference
Gastric	CAFs	IL-6, IL-8, IL-1, IL-22, TGF-β, PGE-2, FGF, TNF-α, and IL-1β, CXCL12	Promotes EMT and tumor invasion	[13,39]
	MSC	WNT5a, Gremlin-1, miR-214, miR-221, and miR-222	Tumor growth and metastasis	[13,40,41]
	TAMs (M2)	EGF, HGF, PDGF, FGF, VEGF, MGF-E8, MCP-1, COX-2/PGE-2, IFN-γ and ROS	Improves cell growth, drug resistance, upregulation of CD44	[13,42,43]
	Endothelial cells	CXCR4	Tumor invasion	[13]
	Myofibroblasts	R-spondin3	Proliferation of Axin2+ Lgr5− stem cells	[13]
	BMDCs	IL-6 and HGF	Increase proliferation and stemness	[43]
Colon	TAMs (M2)	IL-10, PD-1	Immune evasion	[44]
	Tregs	IL-10, TGF-β, PD-L1, PD-L2 and CTLA-4	Immune evasion	[44,45]
	CAFs	TGF-β1, IL-β1, IL-6, IL-33, ROS, C-X-C chemokine receptor (CXC), MMPs, lysyl oxidase, miR-21, TNF-α, and alpha-smooth muscle actin (aSMA), HGF	ECM remodeling, stemness phenotype	[41,46]
	Granulocytes MDSCs	ROS	Induce hypoxic phenotype	[44]
	Endothelial cells	SNAIL, Jagged-1, AKT	Proliferation, stemness, and induce drug resistance	[41,46]
	MSCs	VEGF	Angiogenesis and liver metastasis	[41]
	BMDCs CD34+ CD31−	MMP9, MMP2	Tumor invasion	[41]

**Table 4:** Matrix metalloproteinases in gastrointestinal cancers.

Cancer	MMP	Role in Cancer	Reference
Gastric	2, 1 and 9	Promotes in tumor invasion, especially degradation of the basement membrane	[79]
	13 and MT1-MMP and/or MMP-2	Progression of GC	[80]
	7	Promotes metastasis	[80]
Colorectal	1	Correlates with tumor stage and poor prognosis, level of invasion, lymph node involvement, and metastasis	[79,81]
	2	Correlates with cancer invasion.	[80,81]
	3 and 9	Cancer progression and poor prognosis	[80]
	9	Contributes to inflammation and metastasis	[80,81]
	7	Relates to nodal or distant metastasis, and cell proliferation	[80,81]
	12	Expression reduces tumor growth and increases survival	[81]
	13	Related with advanced cancer stage and poor survival	[80,81]

**Table 5:** ECM deregulated components in colorectal and GC with associated stemness.

ECM Component	Type of Cancer	Role in Cancer Stemness	Clinical Relevance	Ref
Tenascin	Gastric Colorectal	Upregulation of NOTCH ligand, Jagged 1 and other NOTHC components; enhance the expression of LGR5 and MSI1, the WNT and NOTCH signaling	Increased expression in pre-malignant and malignant epithelia	[97,98]
Fibrous protein Collagen type I	Gastric Colorectal	Stemness and tumorigenicity maintenance; increases expression of CD133 and Cmi1. Improve EMT and clonogenicity in CRC CSCs through α2β1 integrin; enhance tumor potential and self-renewal of ALDH+ CSCs through β1 integrin and FAK signaling	Overexpression correlated with overall survival	[99,100,101]
Fibronectin	Gastric Colorectal	FN is a marker of cancer stemness and induces EMT, promote resistance and poor prognosis	Activates intracellular signaling, mediated by integrins, TLRs, Wnt/βcatenin, and P13K, t	[102]
Secreted protein Acidic and Rich in Cysteine (SPARC) Gastric	Colorectal	Associated with EMT	Overexpression better prognosis Overexpression and chemosensitivity Survival prognosis and the clinical features of the tumor were significantly associated with survival, differentiation, and staging.	[103,104]
Periostin (POSTN)	Colorectal Gastric	POSTN promotes stemness and mesenchymal phenotype in human epithelial cells; plays an essential role in the crosstalk between CSCs and the niche leading to metastasis; recruits Wnt ligands, and increases signaling by promoting CSC maintenance and expansion	Correlation with tumor size, grade of cell differentiation, lymph node metastasis, serosal invasion, clinical stage, and 5-year survival rates.	[105,106,107,108]
Biglycan	Colorectal Gastric	Biglycan is highly expressed in colon CSCs and promotes chemoresistance of colon cancer cells by activating NF-kβ signaling	High levels of biglycan are associated with cancer aggressiveness, tumor stage, lymph node metastasis, and worse overall patient survival	[109,110]
Galectin	Colorectal Gastric	Regulated by HIF-1 and it plays vital pro-tumorigenic roles within the tumor microenvironment.	Pathogenesis of gastrointestinal malignancies, favoring tumor development, aggressiveness, metastasis, immunosuppression, and angiogenesis.	[111]

**Table 6:** Target molecules and pathways for gastrointestinal cancer stem cells.

Therapeutic Agent	Inhibitory Mechanism	Mode of Action	Ref
Gemcitabine	EMT	Reduce the frequency of CTC	[136,137]
Apatinib napabucasin (BBI-608), pacritinib	EMT/Angiogenesis	Targeting Jak2/STAT3 block PI3K/AKT and VEGFR2/RAF/MEK/ERK signaling pathways	[138,139,140]
Artesunate	Cell oncosis	β-catenin	[141]
DKN-01	Wnt/β catenin signaling	DKK1	[141,142]
Berberine Metformin	EMT	Smad-independent and Smad-dependent transforming growth factor-β signaling pathway	[143,144]
Genistein	CSCs characteristics by Gli1 signaling pathway.	Tyrosine kinase and topoisomerase II. SFRP2 silencer inhibitor	[141,145]
DS-7423	Apoptosis by p53 induction	PI3K and mTOR	[141,146]
Wogonin	EMT	IL-6/STAT3 signal pathway	[147]
Bigelovin	EMT	N-and E-cadherin, STAT3 pathway, and cofilin pathway	[148]
Cordycepin	EMT. Upregulating cancer cell apoptosis and eliciting cell cycle arrest	Upregulation of CLEC2 via the PI3K/Akt signaling pathway	[149]
Dichloroacetate	Increased responsiveness to 5-FU	PDK-1	[150]
CART-133	Tumor cell apoptosis	CSC CD133+	[151]
Sulfasalazine	CD44v-positive cancer cells	xCT	[152]
LGK974, Foxy-5, PRI-724	Wnt/β-catenin signaling	PORCN inhibitor, WNT5A mimic, β-catenin/CREBBP inhibitor	[153]
Ginsenoside Rg3 combined with cisplatin	TME	EMT	[154]
